# Clinical outcome, long‐term survival and tolerability of sequential therapy of first‐line crizotinib followed by alectinib in advanced ALK+NSCLC: A multicenter retrospective analysis in China

**DOI:** 10.1111/1759-7714.14232

**Published:** 2021-12-01

**Authors:** Zihua Zou, Xuezhi Hao, Cuiying Zhang, Haojing Li, Guilan Dong, Yumei Peng, Kewei Ma, Ye Guo, Li Shan, Yan Zhang, Li Liang, Yangchun Gu, Puyuan Xing, Junling Li

**Affiliations:** ^1^ Department of Medical Oncology, National Cancer Center/National Clinical Research Center for Cancer/Cancer Hospital Chinese Academy of Medical Sciences and Peking Union Medical College Beijing China; ^2^ Cancer Center, Inner Mongolia Autonomous Region People's Hospital Huhhot China; ^3^ Oncology Department Tangshan People’ s Hospital Tangshan China; ^4^ Cancer Center The First Hospital of Jilin University Changchun China; ^5^ Department of Thoracic Oncology Tumor Hospital Affiliated to Xinjiang Medical University Urumqi China; ^6^ Department of Medical Oncology and Radiation Sickness Peking University Third Hospital Beijing China

**Keywords:** Alectinib, ALK+ NSCLC, Crizotinib

## Abstract

**Background:**

There is limited data on the clinical outcome, long‐term survival and tolerability of sequential therapy of first‐line crizotinib followed by alectinib in a real‐world setting for Chinese patients with advanced ALK+ NSCLC.

**Methods:**

The medical records of patients who received sequential therapy with first‐line crizotinib followed by alectinib (no intermittent systemic therapy was allowed between the two ALK‐TKIs) were collected from six centers in China. Combined time treatment to failure (C‐TTF) was defined as the period from the start of crizotinib to the complete discontinuation of alectinib due to any cause.

**Results:**

A total of 61 patients were included in our study. Fifty‐two patients were switched to alectinib due to disease progression, seven as a result of toxicity, and two due to patient preference. At the time of data cutoff, alectinib treatment was discontinued in 31 patients on account of disease progression while severe adverse events resulted in cessation of alectinib in another two patients. Rebiopsy was conducted in 21 patients following disease progression on alectinib in whom *ALK* secondary mutation was found in 13 patients. Patients with *ALK* secondary mutation demonstrated better PFS during treatment with subsequent ALK‐TKIs compared with those without (10.4 vs. 3.1 m, *p* = 0.0018, HR = 0.08). With a median follow‐up of 34.3 months, C‐TTF was 39.2 months and estimated 5‐year OS was 68.6% in the overall population.

**Conclusion:**

Sequential therapy with first‐line crizotinib followed by alectinib demonstrated long‐term benefits. Different efficacy in subsequent ALK‐TKI between patients with or without ALK secondary mutation further emphasized the importance of rebiopsy to guide targeted therapy more precisely.

## Graphical abstract

Sequential therapy with first‐line crizotinib followed by alectinib demonstrated long‐term benefits in a Chinese population. Different efficacy in subsequent ALK‐TKI between patients with or without *ALK* secondary mutation further emphasized the importance of rebiopsy to guide targeted therapy more precisely.

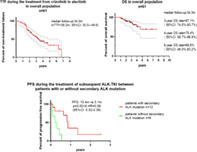

## INTRODUCTION

Lung cancer is the highest cancer‐related cause of mortality worldwide.^1^ Non‐small cell lung cancer (NSCLC) accounts for approximately 85% of lung cancer cases. Patients with advanced NSCLC have experienced a dismal prognosis in the era of chemotherapy. Over the past few decades, there has been huge progress in tumor molecular biology. Several driver gene mutations in NSCLC have been found which have radically transformed the treatment landscape in NSCLC, from the empirical use of chemotherapy to targeted therapy. ALK gene rearrangement is called the “diamond mutation.”^2^, ^3^, ^4^, ^5^ To date, several ALK‐TKIs have been established as standard treatment options, and some real‐world studies have suggested that patients with advanced ALK positive NSCLC may live for approximately 7 years^6^, ^7^ after sequential use of multiple generations of ALK‐TKIs.

Moreover, sequential therapy with first‐line crizotinib followed by alectinib has also been substantiated to demonstrate favorable long‐term benefits both in a clinical trial (J‐ALEX)^8^ and a real‐world study (WJOG 9516L).^7^

However, there is limited data on the clinical outcomes, long‐term survival and tolerability of this treatment strategy in Chinese patients, although the effectiveness of this sequential therapy has been substantiated in a Japanese population. It should also be noted that analysis of treatment failure, resistance mechanism and efficacy during the treatment of subsequent ALK‐TKIs was not conducted in the WJOG 9516L and J‐ALEX studies. More importantly, as CNS is the sanctuary site of crizotinib due to its poor penetration rate across the brain–blood‐barrier (BBB), CNS activity of subsequent ALK‐TKIs is particularly important following the intracranial progression of crizotinib. To date, more clinical practice data is needed to further confirm the intracranial efficacy of alectinib, as patients with symptomatic CNS metastases have been excluded in all alectinib clinical trials^9^, ^10^

Here, we report the clinical outcomes, long‐term survival and tolerability of sequential therapy of first‐line crizotinib followed by alectinib in Chinese patients. Furthermore, we also analyze the resistance mechanism of alectinib and efficacy of subsequent treatment with ALK‐TKIs.

## METHODS

### Patients and data collection

Data of patients diagnosed with advanced ALK+ NSCLC treated with first‐line crizotinib sequential therapy followed by alectinib (no intermittent systemic therapy was allowed between the two ALK‐TKIs) were collected in six hospitals in China from September 2016 to March 2021. Patients with symptomatic or active CNS metastases were included in this study. An MRI scan for intracranial lesions, CT scan for baseline extracranial lesions and during the period of follow‐up were required. Adverse events, reasons for discontinuation of targeted therapy, progression pattern and *ALK* secondary mutation at the progression of alectinib were also recorded. The data cutoff date was June 1, 2021.

### Assessments

The definition and evaluation of intracranial or extracranial lesions were based on the Response Evaluation Criteria in Solid Tumors version 1.1 (RECIST 1.1). In other words, up to five target lesions (≥1 cm) in the whole body and up to two target lesions (≥1 cm) in each organ were included; progression‐free survival (PFS) was calculated from the start of targeted therapy to the date of disease progression. CNS time to progression (CNS TTP) of alectinib was calculated from the start date of alectinib in patients with intracranial lesions until CNS progression. Time to treatment failure (TTF) was defined as the period from the start of targeted therapy to the complete discontinuation of treatment due to any cause including disease progression, death, severe adverse events or patient preference. Combined time to treatment failure (C‐TTF) was defined as the period from the start of crizotinib to the complete discontinuation of alectinib due to any cause. Overall survival (OS) was calculated as the period from the start of crizotinib to the date of death due to any cause.

The extent of improvement in CNS‐related symptoms was mainly based on subjective reports from patients categorized into four different levels (significant improvement, moderate improvement, no improvement, and deterioration). Pleural or pericardial effusion, metastases in the contralateral lung, in nonregional draining lymph nodes or in extrathoracic organs were deemed as distant metastases. Metastases in symmetrical organs such as adrenal glands, or in osseous tissues were considered as one distant organ involved.

### Statistical analysis

Statistical analysis was conducted using SPSS 26.0 statistical software (SPSS, Inc.). Patient distribution and baseline demographic/clinical characteristics are described using frequency analysis. The objective response rate in intra‐ and extracranial lesions was estimated with 95% confidence interval (CI) based on the exact binomial distribution. Differences between groups were compared using the Pearson's χ^2^ test for categorical data, and *t*‐test for continuous data. The survival curves were estimated using the Kaplan–Meier method, while differences in the variables were calculated using the log‐rank test. Cox's proportional hazard model were used to estimate the hazard ratio (HR) and the corresponding 95% CI for the covariate of interests. A two‐sided *p*‐value <0.05 was considered statistically significant.

## RESULTS

### Baseline characteristics during treatment with crizotinib

In total, 61 patients were included in our study. Detailed baseline characteristics during treatment with crizotinib are described in Tables 1 and 2. Patients with ECOG ≥2 points before the administration of crizotinib accounted for nearly one fifth of the overall population. In addition, 87% of patients had distant baseline metastases and metastases in ≥3 distant organs were found in nine patients (14.7%). Ten patients were diagnosed with CNS metastases before crizotinib and only one patient experienced CNS related symptoms. One patient received local treatment for intracranial lesions before first‐line treatment while all patients diagnosed with CNS metastases had uncontrolled baseline CNS lesions (uncontrolled CNS lesions meant either CNS metastases were not treated before or progressed after local treatment). The reasons for discontinuation of crizotinib are shown in Table 3.

**TABLE 1:** Baseline characteristics before initiation of crizotinib (*n* = 61)

Characteristics	Number (percentage)
Gender	
Male	29 (47.5%)
Female	32 (52.5%)
Median age	49 (range 25, 81)
<65	57 (93.4%)
≥65	4 (6.6%)
ECOG 0–1	50 (82.0%)
ECOG ≥2	11 (18.0%)
Smoking history	
Never smoker	47 ((77.0%)
Smoker	14 (23.0%)
Pathology	
Adenocarcinoma	58 (95.1%)
Nonadenocarcinoma	3 (4.9%)
Stage	
III	5 (8.2%)
IV	33 (54.1%)
Recurrence after surgery	23 (37.7%)
CNS metastases	
Yes	10 (16.4%)
No	51 (83.6%)
Distant organs involved	
0	8 (13.1%)
1–2	44 (72.1%)
≥3	9 (14.8%)
Target lesions	
Yes	42 (68.8%)
No	19 (31.1%)
Variants	
Unknown	33
Non EML4‐ALK fusion	3
EML4‐V1	13
EML4‐V3	10
Other EML4 variants	3

**TABLE 2:** Characteristics of CNS metastases during treatment with crizotinib (*n* = 10)

Local treatment before crizotinib	*n* = 1
Uncontrolled CNS lesions before crizotinib	*n* = 10
Symptoms related to CNS metastases before crizotinib	*n* = 1
Measurable intracranial lesions	*n* = 5
Radiological evaluation in CNS during treatment with crizotinib	*n* = 9

**TABLE 3:** Baseline characteristics before the initiation of alectinib (*n* = 61)

	Number (percentage)
Reasons for the discontinuation of crizotinib	
Disease progression	52 (85.2%)
Severe adverse events	7 (11.5%)
Patient preferences	2 (3.3%)
Median age	49 (range 28, 81)
<65	53 (86.9%)
≥65	8 (13.1%)
ECOG	
0–1	40 (65.6%)
≥2	21 (34.4%)
CNS metastases	
Yes	38 (62.3%)
No	23 (37.7%)
Distant organs involved	
0	3 (4.9%)
1–2	38 (62.3%)
≥3	20 (32.8%)
Target lesions	
Yes	46 (75.4%)
No	15 (24.6%)

### Efficacy during treatment with crizotinib

**FIGURE 1:**
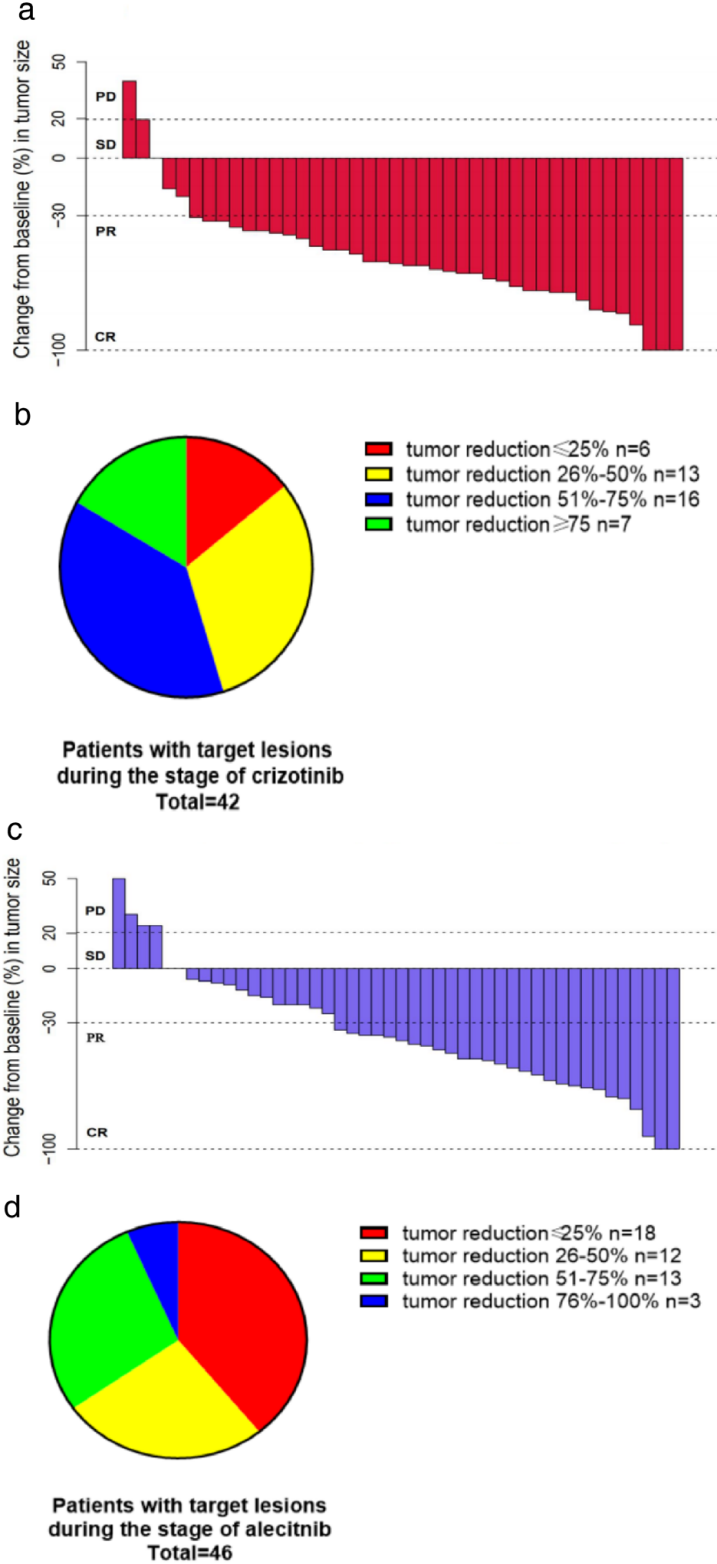
(a) Waterfall plots for patients with target lesions during treatment with crizotinib (*n* = 42). (b) Extent of tumor reduction during treatment with crizotinib (*n* = 42). (c) Waterfall plots for patients with target lesions during treatment with alectinib. (d) Extent of tumor reduction during treatment with alecitnib (*n* = 46)

**FIGURE 2:**
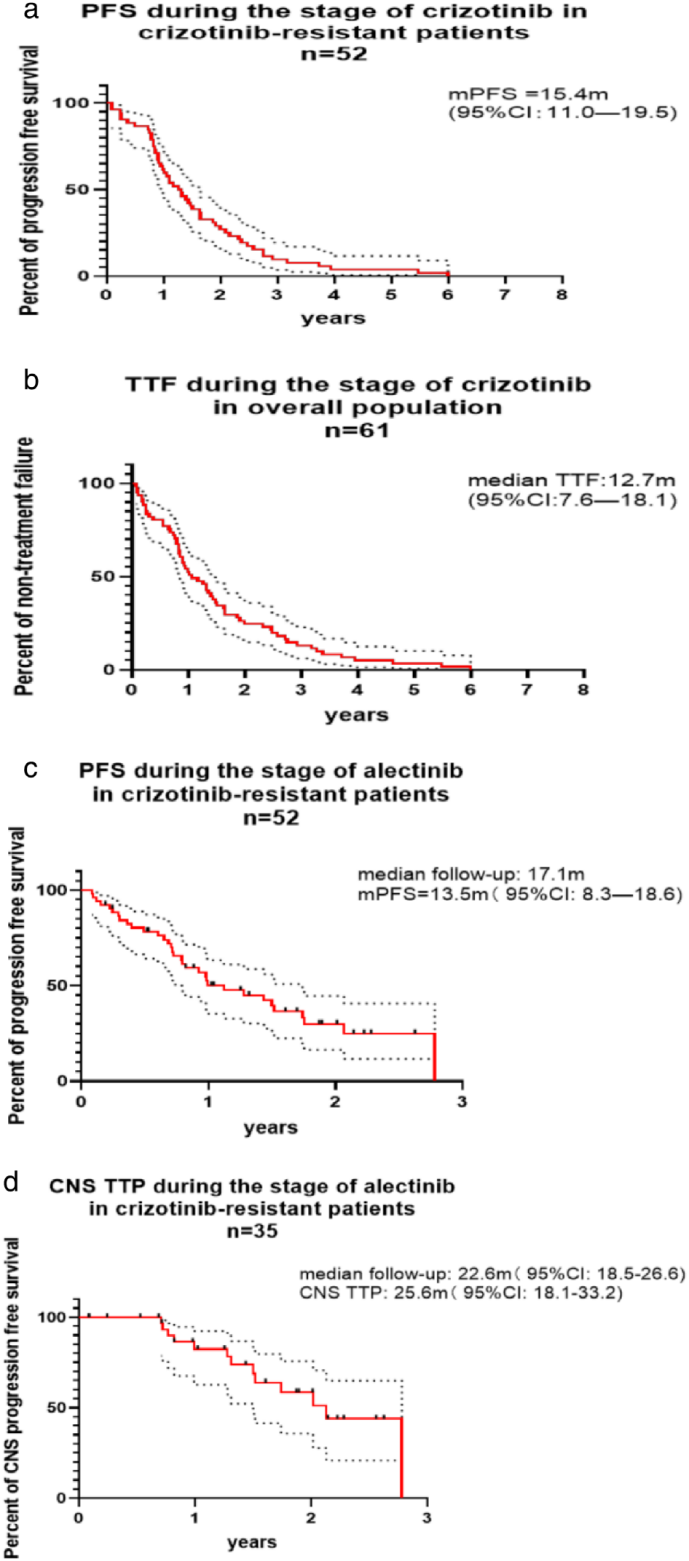
(a) Progression‐free survival during treatment with crizotinib in crizotinib‐resistant patients (*n* = 52). (b) Time to treatment failure during treatment with crizotinib in the overall population (*n* = 61). (c) Progression‐free survival during treatment with alectinib in crizotinib‐resistant patients (*n* = 52). (d) CNS time to progression during treatment with alectinib in crizotinib‐resistant patients (*n* = 35)

One patient received crizotinib only for several days because of intolerable adverse events; therefore, 60 patients had at least one radiological evaluation during first‐line treatment. Overall response rate (ORR) was 60% (1CR + 35PR) in these patients (Table 4) as 85.7% (1CR + 35PR) of patients with target lesions demonstrated a radiological response. Median maximum tumor shrinkage rate was 56% (range −40%, 100%) (Figure 1a) in patients with measurable lesions and over half had tumor reduction over 50% (Figure 1b). As for patients with CNS metastases, intracranial ORR was 22.2% (2PR), whereas 40% of patients (2PR) with measurable CNS lesions were found to have an intracranial response (Table 4). One patient with symptomatic CNS metastases experienced no improvement in symptoms and required salvage brain radiotherapy. Median PFS was 15.4 months (95% CI: 11.1–19.6 m) in crizotinib‐resistant patients (Figure 2a) (*n* = 52) in whom 22 patients developed progression only in CNS, while 18 patients experienced extracranial progression. Twelve patients were reported to have progression both in intra‐ and extracranial lesions.

**TABLE 4:** Efficacy during treatment with crizotinib

ORR in patients with at least one radiological evaluation (*n* = 60)	60% (1CR + 35PR) (95% CI: 46.5%–72.4%)
ORR in patients with target lesions (*n* = 42)	85.7% (1CR + 35PR) (95% CI: 71.5%–94.6%)
Median maximum tumor reduction rate (*n* = 42)	56% (range: −40%, 100%)
Intracranial ORR in patient with CNS metastases following at least one radiological evaluation (*n* = 9)	22.2% (2PR) (95% CI: 2.8%–60.0%)
Intracranial ORR in patient with measurable CNS lesions (*n* = 5)	40% (2PR) (95% CI: 5.3%–85.3%)
Improvement in CNS related symptoms (*n* = 1)	
Significant improvement	0
Moderate improvement	0
No improvement	1
Deterioration	0

### Safety of crizotinib

A total of 57 patients had detailed safety records during the treatment of crizotinib, and common adverse events are listed in Table S1. The majority of patients experienced grade 1–2 adverse events with grade 3–4 adverse events found in 12.3% of patients. No symptomatic bradycardia, ≥grade 2 elongation in QTc interval and interstitial pneumonia were reported. Dose interruption was observed in 24.6% of patients and grade 2–4 elevation in transaminoferase (10 patients) was the most common reason. A total of 15.8% of patients were reported to have at least one dose reduction while seven patients had permanently discontinued crizotinib treatment in whom five patients experienced grade 3–4 elevation in transaminoferase.

### Time to treatment failure during treatment with crizotinib

A total of 52 patients were switched to treatment with alectinib due to disease progression, while criztonib was completely discontinued in seven patients because of severe adverse events and the treatment option was changed in another two patients on account of their own preferences. Median TTF was 12.7 months (95% CI: 7.4–17.9 m) in the overall population (Figure 2b).

### Baseline characteristics during treatment with alectinib

The number of patients with intracranial metastases rose to 38 before the initiation of alectinib with symptomatic CNS metastases reported in 10 patients (Tables 3 and 5). Seven patients underwent local treatment for CNS lesions at intracranial progression of crizotinib while over 90% of patients were found to have uncontrolled CNS metastases before treatment with alectinib. Patients with ECOG ≥2 points accounted for over 30% of overall population before administration of alectinib.

**TABLE 5:** Characteristics of CNS metastases before initiation of alectinib (*n* = 38)

Intracranial characteristics	Number (percentage)
Local treatment before alectinib	
Yes	7 (18.4%)
No	31 (81.6%)
Uncontrolled CNS metastases	
Yes	36 (94.7%)
No	2 (5.3%)
Symptoms related to CNS lesions	
Yes	10 (26.3%)
No	28 (73.7%)
Measurable CNS metastases	
Yes	19 (50.0%)
No	19 (50.0%)

### Efficacy during treatment with alectinib

All patients included in the study had at least one radiological evaluation, and ORR in the overall population was 47.5% (2CR + 27PR) (Table 6). A radiological response was found in 28 patients (60.9%, 1CR + 27PR) with target lesions. Median maximum tumor shrinkage rate was 39% (Figure 1c) as over 30% of patients with measurable lesions were found to have tumor reduction of over 50% (Figure 1d). A total of 47.4% of patients with CNS metastases demonstrated an intracranial response while intracranial ORR was 68.4% in patients with measurable CNS lesions (Table 6). As for patients with symptomatic CNS metastases, nine patients were found to experience significant improvement in CNS‐related symptoms and no patient was in need of further salvage brain radiotherapy. PFS in crizotinib‐resistant patients during treatment with alectinib was 13.5 months (95% CI: 8.3–18.6) with median follow‐up of 17.1 months (95% CI: 8.9–26.1) (Figure 2c). CNS TTP of alectinib was 25.6 months (95% CI: 18.1–33.2) in crizotinib‐resistant patients with median follow‐up of 22.6 months (95% CI: 18.5–22.6) (Figure 2d) (38 patients had CNS metastases before the administration of alectinib, in whom three patients were switched to alectinib due to adverse events).

**TABLE 6:** Efficacy during treatment with alecitnib

ORR in overall population (*n* = 61)	47.5% (2CR + 27PR) (95% CI: 34.6%–60.7%)
ORR in patients with target lesions (*n* = 46)	ORR = 60.9% (1CR + 27PR) (95% CI: 45.4%–74.9%)
Median maximum tumor shrinkage rate (*n* = 46)	39% (range: −50%, 100)
Intracranial ORR in patients with CNS metastases (*n* = 38)	47.4% (7CR + 11PR) (95% CI: 31.0%–64.2%)
Intracranial ORR in patients with measurable CNS lesions (*n* = 29)	68.4% (2CR + 11PR) (95% CI: 43.4%–87.4%)
Improvement in CNS related symptoms (*n* = 10)	
Significant improvement	9 (90%)
Moderate improvement	1 (10%)
No improvement	0
Deterioration	0

### Safety of alectinib

A total of 56 patients had detailed safety records during treatment with alectinib. Table S1 shows the common adverse events following treatment with alectinib. Most patients were reported to have grade 1–2 adverse events while only two patients experienced severe adverse events. A total of 17.8% of patients (*n* = 10) had at least one dose interruption with a total bilirubin increase being the most common reason. Dose reduction was reported in 8.9% of patients (*n* = 5) as total bilirubin increased and elevation in creatine kinase contributed most to dose adjustments. No symptomatic bradycardia or ≥grade 2 elongation in QTc interval were found. Grade 5 interstitial pneumonia and grade 4 total bilirubin increase, which resulted in permanent discontinuation of alectinib, were recorded in two patients, respectively.

### Progression pattern, resistance mechanism and subsequent therapy at the progression of alectinib

At the time of data cutoff, 36 patients developed progression during treatment with alectinib. CNS progression was reported in 11 patients, 22 patients experienced extracranial progression and three patients were found to develop intra‐ and extracranial lesion progression. Rebiopsy was conducted in 21 patients in whom secondary mutation in ALK kinase domain was found in 13 patients, and the specific mutation site is described in Figure 4a. Nine patients with local or gradual progression continued alectinib treatment as well as other local treatment or antiangiogenic agents. As for patients in whom treatment with alectinib had been discontinued, 87.9% (29/33) of patients underwent more than one line of subsequent therapy as 27 patients received further treatment with other ALK‐TKIs.

### Time to treatment failure during treatment with alectinib

At the time of data cutoff, treatment with alectinib had been discontinued in 33 patients and 31 patients had switched to other treatments due to disease progression, while severe adverse events resulted in the cessation of treatment with alectinib in two patients (Table S1). With median follow‐up of 18.6 months, TTF during treatment with alectinib was 17.2 months (95% CI: 8.9–26.1 m) in the overall population (data was not shown in the Figure).

### Overall survival and combined time to treatment failure

**FIGURE 3:**
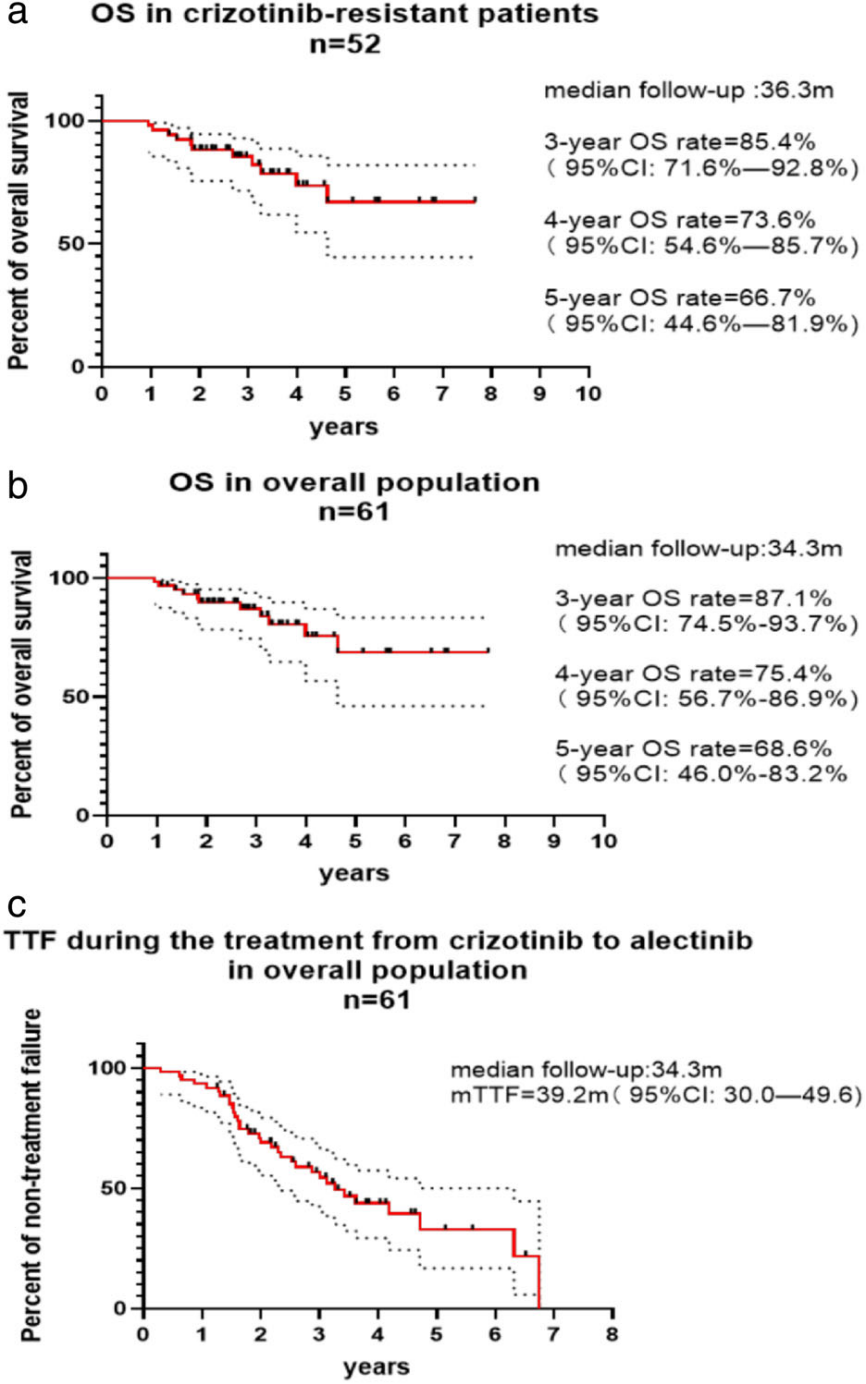
(a) Overall survival in crizotinib‐resistant patients (*n* = 52). (b) Overall survival in overall population (*n* = 61). (c) Time to treatment failure during treatment with crizotinib to alectinib in the overall population (*n* = 61)

For crizotinib‐resistant patients, the median OS was not reached as 3‐year survival rate was 85.4% (95% CI: 71.6%–92.8%), estimated 4‐year survival rate was 73.6% (95% CI: 54.6%–85.7%), estimated 5‐year survival rate was 66.7% (95% CI: 44.6%–81.9%) with median follow‐up of 36.3 months (95% CI: 29.7–42.8 m) (Figure 3a). In the overall population, duration of median follow‐up was 34.3 months (95% CI: 28.9–39.6 m), 3‐year survival rate was 87.1% (95% CI: 74.5%–93.7%), estimated 4‐year survival rate was 75.4% (95% CI: 56.7%–86.9%), estimated 5‐year survival rate was 68.6% (95% CI: 46.0%–83.2%) (Figure 3b) and C‐TTF was 39.2 months (95% CI: 30.0–49.6 m) (Figure 3c).

### Univariate and multivariate analysis of PFS for crizotinib‐resistant patients

Univariate analysis of PFS during treatment with crizotinib is shown in Table 7, and covariates with *p* < 0.1 in the univariate analysis are included in the Cox model. Results from multivariate analysis suggested that females experienced superior PFS to males (*p* = 0.010, HR = 0.454, 95% CI: 0.248–0.830) and patients with worse ECOG (≥2 points) had more unfavorable PFS compared with their counterparts (*p* = 0.006, HR = 2.811, 95% CI: 1.355–5.835). Univariate analysis of PFS during treatment with alectinib is shown in Table 8, and likewise covariates with *p* < 0.1 in the univariate analysis are included in the Cox model. Although no significant predictive factor was found in the multivariate model, patients who experienced extracranial progression or intracranial plus extracranial progression simultaneously seemed to demonstrate worse PFS compared with those who only developed progression in CNS following treatment with crizotinib (*p* = 0.062 [borderline], HR = 2.509, 95% CI: 0.955–6.592).

**TABLE 7:** Predictive factors for PFS of crizotinib in crizotinib‐resistant patients (*n* = 52)

Variable	Univariable analysis, *p*‐value	Multivariable analysis	
		Hazard ratio (95% CI)	*p*‐value
Age		HR = 2.063	*p* = 0.211
≥65 vs. <65	0.05	(95% CI: 0.663–6.417)	
Gender		HR = 0.454	*p* = 0.010
Female vs. male	0.003	(95% CI: 0.248–0.830)	
ECOG		HR = 2.811	*p* = 0.006
≥2 vs. 0–1	0.003	(95% CI: 1.355–5.835)	
Smoking history			
Smoker vs. never smoker	0.392	‐	
Stage			
III or recurrence without distant metastases			
vs.	0.475	‐	
IV or recurrence with distant metastases			
Distant organs involved		HR = 1.883	*p* = 0.115
≥3 vs. ≤2	0.077	(95% CI: 0.857–4.137)	
CNS metastases			
Yes vs. no	0.671	‐	

**TABLE 8:** Predictive factors for PFS of alectinib in crizotinib‐resistant patients (*n* = 52)

Variable	Univariable analysis, *p*	Multivariable analysis	
		Hazard ratio (95% CI)	*p*‐value
Age			
≥65 vs. <65	*p* = 0.51	‐	
Gender			
Female vs. male	*p* = 0.299	‐	
ECOG		HR = 1.681	*p* = 0.161
≥2 vs. 0–1	*p* = 0.056	(95% CI:0.813–3.474)	
Smoking history			
Smoker vs. never smoker	*p* = 0.675	‐	
Distant organs involved			
≥3 vs. ≤2	*p* = 0.676	‐	
CNS metastases		HR = 0.492	*p* = 0.124
Yes vs. no	*p* = 0.001	(95% CI: 0.199–1.124)	
Progression pattern of previous crizotinib			
Extracranial progression or extracranial + CNS progression		HR = 2.509	*p* = 0.062
vs.		(95% CI: 0.955–6.592)	
CNS progression	*p* = 0.001		
PFS of previous crizotinib			
<12 vs. ≥12 m	*p* = 0.145		

### Impact of EML4‐variants on PFS during treatment with crizotinib

This section of analysis was performed in crizotinib‐resistant patients with known types of EML4‐ALK variants (25 patients with EML4‐ALK fusion, V3 found in 12 patients, EML4‐non V3 found in 13 patients, one patient with V3 and one patient with V1 switched to alectinib due to adverse event or preference, these two patients were excluded in this part of analysis). The baseline characteristics listed in Table S2 as important features were all balanced between patients with EML4‐V3 and EML4‐non‐V3. Our results indicated that patients carrying variant three demonstrated numerically shorter PFS to those with EML4‐non V3 (11.7 vs. 21.3 m, *p* = 0.14, HR = 1.78, 95% CI: 0.75–4.23) (Figure S1).

### Impact of ALK secondary mutation on the efficacy of subsequent ALK‐TKI

**FIGURE 4:**
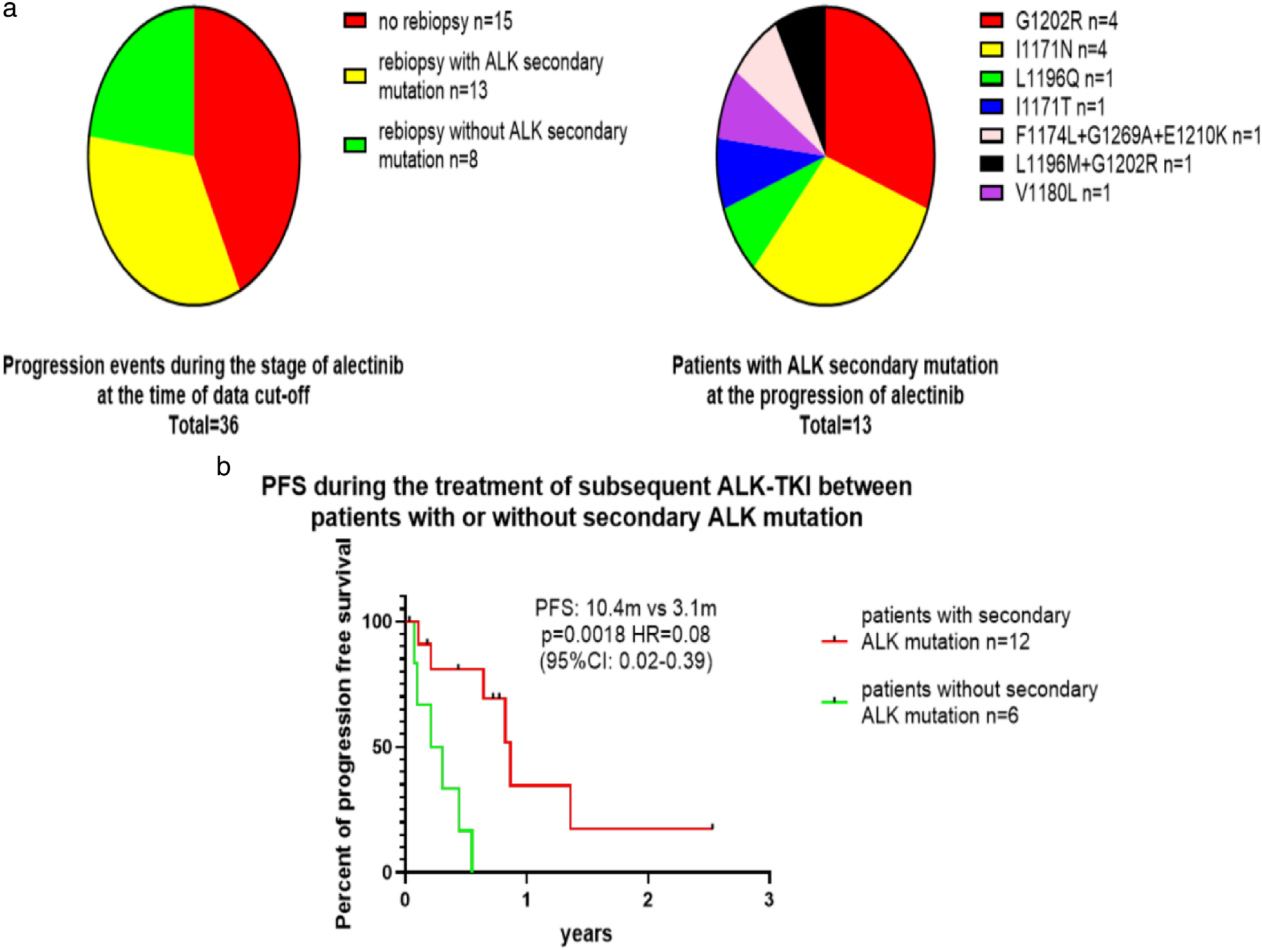
(a) *ALK* secondary mutation following disease progression on alectinib. (b) Progression‐free survival during the treatment of subsequent ALK‐TKI between patients with or without secondary *ALK* mutation

Twelve patients who carried secondary mutation in the ALK kinase domain received subsequent treatment with other ALK‐TKIs, while six patients without ALK resistance mutation underwent unselected use of other ALK‐TKIs (Figure S2 describes detailed information of subsequent ALK‐TKIs). We observed that patients with ALK secondary mutation demonstrated more favorable PFS during the treatment of subsequent ALK‐TKI compared with those without (10.4 vs. 3.1 m, *p* = 0.0018, HR = 0.08, 95% CI: 0.016–0.389) (Figure 4).

## DISCUSSION

Several ALK‐TKIs have previously been developed as standard treatment for patients with advanced ALK+NSCLC. Sequential use of multiple generations of ALK‐TKIs has demonstrated favorable long‐term benefits, which have been firmly confirmed in clinical trials or in real‐world studies.^6^, ^7^, ^8^ Sequential therapy with crizotinib followed by alectinib is widely used in clinical practice; however, further analysis for this treatment strategy is still needed. For example, reasons for treatment failure were not specifically described in the WJOG 9516L study; moreover, patients with symptomatic CNS metastases were excluded in all alectinib clinical trials, therefore CNS efficacy of alectinib for these is in need of further investigation in a real‐world setting.

Here, we first report the clinical outcomes, long‐term survival and tolerability of sequential therapy of crizotinib followed by alectinib in a Chinese population. Our results are similar to previous findings which further substantiate the clinical value of this sequential treatment. We observed that the proportion of patients with CNS metastases rose by over 50% during disease progression on crizotinib, which further demonstrates the unfavorable CNS protective effect of crizotinib.^11^ Furthermore, our research indicated that alectinib showed potent CNS activity and brought about significant improvement in CNS related symptoms, which is also consistent with the results of previous studies.^12^ Moreover, c‐TTF in our study and the WJOG 9516L report both reached approximately three years, further suggesting that treatment duration of targeted therapy could be prolonged for patients with local or gradual progression in company with local treatment or antiangiogenic angents. In addition, *ALK* secondary mutation was identified in over 60% of patients who underwent rebiopsy following progression on alectinib, which is also in line with previous findings.^13^ In addition, patients with *ALK* secondary mutation demonstrated superior PFS in subsequent treatment of other ALK‐TKIs compared with those without, which could be explained by the “off‐target” effect of ALK‐TKI previously reported.^14^ We also analyzed the reason for treatment failure, and close heed should be paid to liver toxicity which is often seen in patients who permanently discontinue targeted therapy in clinical practice.

Additionally, we also analyzed the PFS predictive factors of crizotinib and alectinib. During treatment with crizotinib, gender and performance status were the predictive factors of PFS, and poor ECOG was also confirmed as a prognostic factor in a previous study,^15^ while the reason of sexuality to cause an apparent discrepancy in PFS was unknown. During treatment with alectinib, performance status was not an influential factor to PFS, which could be possibly explained by the potent efficacy of alectinib in patients with symptomatic CNS metastases who experienced unfavorable baseline performance status; we also found that patients who develop progression only in CNS following crizotinib treatment appeared to show better PFS compared with those who had extracranial progression or extra‐ plus intracranial progression. The plausible explanation for this phenomenon might be that inadequate CNS exposure was the explicit explanation for intracranial progression of crizotinib^16^ which could be well resolved by alectinib while resistance mechanisms were more complicated for patients who experienced extracranial progression or extracranial plus intracranial progression which could not be overcome by alectinib. Last but not least, our study suggested that patients with V3 appeared to demonstrate worse PFS of crizotinib to those with EML4‐non V3. Incongruent conclusions were drawn from previous studies which investigated the impacts of different ALK variants on the efficacy of crizotinib. Some scholars deemed that V3 with more invasive tumor biology presented a worse response to crizotinib,^17^, ^18^ while some findings indicated that patients with V1 had more favorable PFS of crizotinib compared with non‐V1^19^ and other studies suggested V2 had best response to crizotinib among ALK variants.^20^ Although much progression had been made in this area, some disadvantages still exist in those studies previously mentioned; for example, patients included in some studies received different lines of crizotinib and some studies did not compare the baseline characteristics among patients with different variants. We overcame these shortcomings so as to draw a more compelling conclusion.

Our study had some limitations and many questions still need to be resolved in the future. First, no comparative group was established in our study which inevitably caused a selection bias. In addition, there were fewer patients with baseline CNS metastases and insufficient duration of follow‐up may have meant that the long‐term survival of patients in our study was overestimated. Furthermore, although our results indeed revealed excellent intracranial efficacy of alectinib, we were still unable to reach a clear conclusion as to whether alectinib could lower or defer the need for brain radiotherapy as we failed to discuss the optimal timing of RT in our study. Meanwhile, improvement in CNS‐related symptoms was based on subjective assessments by patients rather than objective parameters. In addition, most patients did not undergo rebiopsy following disease progression on crizotinib; therefore, it could not be fully confirmed whether *ALK* secondary mutation appeared after crizotinib resistance or at disease progression on alectinib. However, given that most patients with *ALK* secondary mutation experienced CNS progression following treatment with crizotinib, we concluded that *ALK* secondary mutation occurred during disease progression on alectinib in our study. Moreover, we did not exclude the influence of comutation such as TP53 when analyzing the impacts of different variants on PFS. Last but not least, whether upfront use of alectinib or sequential treatment of crizotinib followed by alectinib demonstrates superior OS must be resolved in the future. The ALEX study^3^ indicated that OS was elevated by over 20% in patients in the alectinib group compared with those in the crizotinib group. Nonetheless, it should be noted that only half of the patients from the crizotinib group received subsequent ALK‐TKIs, and that the duration of follow‐up in the crizotinib group was only 23 months. In the WJOG 9516L^7^ and J‐ALEX studies,^8^ patients in the sequential therapy group demonstrated similar long‐term survival compared with those who received upfront alectinib.

In conclusion, sequential therapy with first‐line crizotinib followed by alectinib showed survival benefits for patients with advanced ALK+ NSCLC. Different efficacy in subsequent ALK‐TKIs between patients with or without ALK secondary mutation further emphasized the importance of rebiopsy to guide targeted therapy more precisely.

## CONFLICT OF INTEREST

No authors report any conflict of interest.

## Supporting information


**Table S1**: (a) Common adverse events of crizotinib for patients with detailed safety records (n = 57). (b): Outcomes of adverse events during treatment with crizotinib (*n* = 57)
**Table S2**: Baseline characteristics between crizotinib‐resistant patients with EML‐V3 and EML4‐non‐V3
**Figure S1**: PFS during treatment with crizotinib between patients with V3 and EML4‐non‐V3
**Figure S2**: subsequent ALK‐TKI in patients with or without ALK secondary mutation
