# Supplementary material for: Clinical outcome, long‐term survival and tolerability of sequential therapy of first‐line crizotinib followed by alectinib in advanced ALK+NSCLC: A multicenter retrospective analysis in China
Source: Thorac Cancer. 2021 Dec 1;13(1):107–16. doi: 10.1111/1759-7714.14232 (PMC8720624; doi:10.1111/1759-7714.14232)
Supplement: Supplementary file 1 — Table S1: (a) Common adverse events of crizotinib for patients with detailed safety records (n = 57). (b): Outcomes of adverse events during treatment with crizotinib (n = 57) Table S2: Baseline characteristics between crizotinib‐resistant patients with EML‐V3 and EML4‐non‐V3 Figure S1: PFS during treatment with crizotinib between patients with V3 and EML4‐non‐V3 Figure S2: subsequent ALK‐TKI in patients with or without ALK secondary mutation [file TCA-13-107-s001.docx]

**Supplementary Table 1a: Common adverse events of crizotinib for patients with detailed safety records (n=57)**

|  | Grade 1-2 | Grade 3-4 |
| --- | --- | --- |
| Constipation | 14% | 0 |
| Diarrhea | 42.2% | 5.2% |
| Nausea | 47.3% | 1.7% |
| Vomiting | 21% | 0 |
| Edema | 43.8% | 0 |
| Aminotrasferase increased | 40.3% | 8.7% |
| Vision impaired | 29.8% | 0 |
| Rash | 10.5% | 0 |

**Supplementary Table 1b: Outcomes of adverse events during the stage of crizotinib(n=57)**

| Grade 3-4 adverse events | 7(12.3%) |
| --- | --- |
| Dose interruption | 14(24.6%) |
| Common adverse events led to dose interruption | Grade 2-4 aminotransferase increased  n=10 |
| Dose reduction | 9(15.8%) |
| Common adverse events led to dose reduction | Grade 2-4 aminotransferase increased  n=7 |
| Permanent discontinuation due to adverse events | 7(12.3%) |
| Severe adverse events led to permanent discontinuation of crizotinib | Grade 3-4 aminotransferase increased  n=5  Repeated grade 3 diarrhea n=2 |

**Supplementary Table 2: Baseline characteristics between crizotinib-resistant patients with EML-V3 and EML4-non-V3**

|  | EML4-V3 n=11 | EML4-non-V3 n=12 | P value |
| --- | --- | --- | --- |
| Gender  Male  Female | 2(18.2%)  9(81.8%) | 7(58.3%)  5(41.7%) | P<0.05 |
| Age  ＜65  ≥65 | 10(90.9%)  1(9.1%) | 12(100%)  0(0%) | Not sig |
| Pathology  Adenocarcinoma  Non-adenocarcinoma | 11(100%)  0(0%) | 12(100%)  0(0%) | Not sig |
| Smoking history  Never smoker  Smoker | 9(81.8%)  2(18.2%) | 8(66.7%)  4(33.3%) | Not sig |
| Performance status  ECOG 0-1  ECOG ≥2 | 10(90.9%)  1(9.1%) | 10(83.3%)  2(16.7%) | Not sig |
| Stage  III or recurrence without distant metastases  IV or recurrence with distant metastases | 2(18.2%)  9(81.8%) | 0(0%)  12(100%) | Not sig |
| CNS metastases  Yes  No | 0(0%)  11(100%) | 1(8.3%)  12(91.7%) | Not sig |
| Distant organs involved  ≤2  ≥3 | 10(90.9%)  1(9.1%) | 10(83.3%)  2(16.7%) | Not sig |
